# PAD: A graphical and numerical enhancement of structural coding to facilitate thematic analysis of a literature corpus

**DOI:** 10.1016/j.mex.2022.101633

**Published:** 2022-02-15

**Authors:** Etienne-Victor Depasquale, Humaira Abdul Salam, Franco Davoli

**Affiliations:** a Department of Communications and Computer Engineering, Faculty of ICT, University of Malta, MSD2080, Msida, Malta; b High-Energy Physics Research (FH) IT' Group At Deutsches Elektronen-Synchrotron (DESY), Hamburg 22607, Germany; c Department of Electrical, Electronic and Telecommunications Engineering and Naval Architecture (DITEN), The National Laboratory of Smart and Secure Networks (S2N), Italian National Consortium for Telecommunications (CNIT), University of Genoa, Genoa 16145, Italy

**Keywords:** Systematic review, Structural coding, Graph theory

## Abstract

We suggest an enhancement to structural coding through the use of (a) causally bound codes, (b) basic constructs of graph theory and (c) statistics. As is the norm with structural coding, the codes are collected into categories. The categories are represented by nodes (graph theory). The causality is illustrated through links (graph theory) between the nodes and the entire set of linked nodes is collected into a single directed acyclic graph. The number of occurrences of the nodes and the links provide the input required to analyze relative frequency of occurrence, as well as opening a scope for further statistical analysis. While our raw data was a corpus of literature from a specific discipline, this enhancement is accessible to any qualitative analysis that recognizes causality in its structural codes.

Through our work, we claim:

• To extend the semantic potential of structural coding, where the structural codes are causally related, and
• To extend the methodological scope of systematic review.

## Graphical abstract

**Figure:**
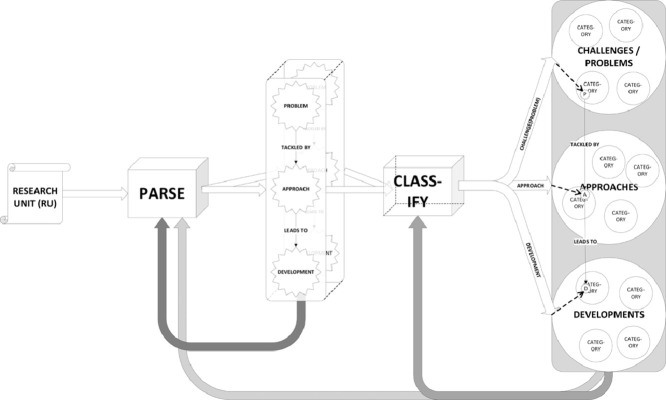

Specification Table

**Table:** 

Subject Area:	Computer science
More specific subject area:	Systematic review
Method name:	PAD: a protocol for systematic review
Name and reference of original method:	Structural coding. Reference: There are many. An example is shown in this table cell. J. Saldana, “An introduction to codes and coding,” in The Coding Manual for Qualitative Researchers, 1st, California: Sage Publications, Inc, 2009, ISBN: 978–1–84,787–549–5.
Resource availability:	https://data.mendeley.com/v1/datasets/gzfbzfgk79

## Motivation

The undertaking of a review is subject to the risk of degenerating into a ramble around the corpus of literature in scope. The SALSA (Search, AppraisaL, Synthesize, Analyze) framework [1] is a significant milestone on the path towards “an internationally agreed set of discrete, coherent and mutually exclusive review types” [1, p. 104] and, thereupon, to the development of a common body of methods for producing reviews. For example, in [2], PSALSAR (Research Protocol, - SALSA, Report Results) adopts, implements and extends SALSA for the type of review that is recognized as systematic literature review.

Tables are a synthetical technique common to several types of review [1, p. 95]. The use of data structures, such as tables and lists, gives all stakeholders a sense of value, but these summaries pose a different problem: each such data structure (the tables, lists and other condensing representations of the corpus) represents a single perspective on the corpus. While these may very well be highly informative and of great value, they fall short in the overarching objective of the survey: a holistic abstraction of the literature that succinctly presents the state of knowledge on the field of study.

This problem may be tackled through the use of analytical methods that are recognized as leading to such an abstraction. ***Thematic analysis*** is one such method, which we have used in surveying, and justify our selection of this form of analysis on:

1. The coherence amongst widely-cited texts [3], [4], [5], [6], [7] in recommending thematic analysis as an introduction to the methods of qualitative analysis, and
2. Its application in [8] to a corpus of literature from the discipline of Computer Science.

However, notwithstanding themes’ abstractive and holistic potential, unless the procedure through which they are obtained is scrutinizable and objective, doubts about the reviewer's bias and skill will tinge the themes. In this paper, through the use of research questions crafted with these two criteria in mind, we propose an enhancement of structural coding (*en route* to thematic analysis of a literature corpus) that converts text into:

• ***Numeric data***, which is then processed using metrics (Section 8) that normalize dataset sizes and thereby lead to frequency analysis;
• ***Graphical objects*** (Section 7), which are then organized into graphical overviews.

These facilitate both objectivity in, and critique of, the insights obtained from the thematic analysis.

## Background and overview

Before we proceed to the mechanics of the method, we summarize the theory out of which this work emerged and lay out the method's principal elements against this backdrop.

We follow the ***codes-to-theory model*** for qualitative inquiry [7, pp. 8–13] and instantiate the model in a form that is suited to the pursuit of a literature survey. The data corpus, as with any literature survey, is none less than the entire body of literature within the survey's scope, and each publication within the corpus is a single item of the textual data. Clearly, careful scope management is a prerequisite to obtaining feasible limits on the number of publications.

While every surveyor will determine his or her own boundaries within a discipline's literature, it is nevertheless useful to distinguish a generalization that cuts across disciplines. Namely, there is a prominent and recognizable type of publication, which documents the pursuit of solving a problem (or challenge) that was hitherto at least partially open within its framing discipline. Some approach, or set of approaches, will constitute the pursuit and, at its end, it should at least have progressed towards one major development and possibly several corollary and/or ancillary ones. Our principal contribution emerges from the observation that ***this dynamic is codable***. It comprises a three-stage motion from problem/challenge, to approach, to development. These stages are describable through pithy codes that pack meaning: they inform the reader through a high-level summary of the entire movement from problem, through approach(es) and onto development(s). Note that meaning is not limited to that obtained from the stages. Indeed, more importantly, it is obtained from the links between the stages, as these reveal the proceedings of research as it unfolds from beginning to end.

Now, “the excellence of the research [(qualitative analysis)] rests in large part on the excellence of the coding” [9, p. 27]. Therefore, choice of coding method requires careful consideration of ***its fitness for the purpose of arriving at the desired abstraction***. We suggest that one highly desirable abstraction would be a set of themes, sufficiently justified, that shows the ***maturity*** of areas explored in the corpus. The issue of finding suitable attributes of maturity elegantly converges with the codable dynamic: the frequency of the uncovered problems, approaches and developments serves as an indicator of maturity. We thus seek research questions crafted to elicit answers, healthily balanced between concision and clarity, that describe these stages for each publication. These answers, or understandable abridgements thereof, ***are the codes***. The questions should be standardized – i.e., applicable to any publication in the corpus – to support comparability of the codes. This coding method is readily recognizable as ***structural coding.*** Our first enhancement of structural coding now follows. To each code, we associate a unique numeric identifier and define the object thus formed out of the association between code and identifier, as a ***node***.

While the outcome of coding a publication in this manner has evident recapitulative value, it falls short of the desired abstraction of the corpus. Furthermore, a repetition of the process over the publications in scope, produces a set of stage-codes that is potentially several times more numerous than the number of publications! Rationalization is desirable, and the formalisation at hand in the codes-to-theory model for qualitative inquiry [7, pp. 8–13] is well suited to this end. The meaning packed into the codes is cognitively unpacked during attempts to proximate codes ***according to meaning*** into ***categories***. The categories thus formed are bound to the stages. Problem categories, approach categories and development categories respectively circumscribe problem codes, approach codes and development codes by generalizing a core aspect of the gathered codes. As was done with the code, each category is transformed by binding it to a unique numeric identifier and encapsulating code and identifier in a ***category-node***. The speed with which such categorization proceeds depends on the surveyor, as it hinges rigidly upon a framework of knowledge of the discipline, and the surveyor may be in the very process of developing this knowledge. Even an expert surveyor can expect to add codes, re-write them to facilitate categorization and remove those that turn out to be marginal or trivial in the whole schema. This is the ***cyclical*** [7, p. 8] nature of coding.

As categories are formed out of the proximal codes, a re-validation of the links between the codes is necessary to ensure that they still hold as links between the holding categories. For example, consider two problem-stage codes that are linked to a common approach code. If the codes are grouped into a category, the two links, one per problem-stage code, are ***aggregated***. This new aggregate must be interpreted by the surveyor for meaning that applies to the individual links in the aggregate. Just as categorization gathers codes, so does it aggregate links obtained with the diversity of the code level, and assist the surveyor achieve the ultimate goal of the survey.

All the necessary elements have now been exposed and we may proceed to unify them into a ***causal network***. There are (category-)nodes; there are interconnecting links that represent the causality that inheres in the researcher's urge to deal with a challenge through some set of approaches and therefrom obtain developments (through analysis of the results). This causal network is a ***directed acyclic graph*** (DAG) and it is amenable to statistical analysis. Perhaps most satisfyingly, it is also fruitful under visual inspection. ***Minimally***, we expect the frequency of problems, approaches and developments to contribute to themes. A better understanding of ***maturity*** would be the overarching goal of these themes. Moreover, we expect the frequency of occurrence of the links, notably that between problem category nodes and approach category nodes, to shed light on the ***fecundity*** of specific pathways in the overall motion of research in the discipline. The pathways may be either two-stage (P node – A node / A node – D node) and we refer to them as ***dyads***, or they may be three-stage (P node – A node – D node) and we refer to them as ***triads***. Thus, if the frequency of occurrence of links in the pathways (dyads or triads) is represented through their thickness, then such insights are gained simply by inspection of the DAG.

Further still, a more liberal characterization of the corpus is possible simply through the facility afforded by the processes of coding and categorization. Such processes build knowledge and facilitate critical analysis of publications’ approaches and developments. For example, this supports the development of critique that warns against pitfalls and fallacies. Generally, any pattern that emerges through coding's repetitiveness and categorization's summarizing facility, is a valid candidate for the final set of themes.

## Contents

1. In Section 4, the concept of the "code" is revisited (Section 4.1). We declare our use of ***structural coding*** as the fundamental coding technique and specify the series of questions applied to the raw data (Section 4.2). This is followed by an explanation of the reasoning that guided the extraction of the codes (Section 4.3).
2. In Section 5, we introduce the ***node*** and ***link*** concepts from graph theory (Section 5.1) and proceed to relate the ***causally-bound codes*** to the node and link. An algorithm for categorization is presented (Section 5.2). We then extend the causal linkage inherent to the mined codes to the resulting categories.
3. Section 6 develops the application of nodes in this method by defining attributes that support further processing of the nodes.
4. In Section 7, the graphical means for communicating a survey's results are described.
5. Statistics produced by the method, as well as intuition on their significance, are described in Section 8.
6. An explanation of how the method facilitates thematic analysis is presented in Section 9.
7. Observations on the application of the method are presented through case notes in Section 10, where we draw an outline of how this method was applied to a recent work.
8. We conclude by identifying benefits and limitations (Section 11.1) of the method and summarize the prescriptions of the method in Section 11.2.

## Coding: the core technique

### What are codes?

**Codes are terse, dense, textual representations of a verbose articulation of a concept embedded in the raw data**. The prescriptions of sound qualitative analysis for systematic review require a choice of ***coding method***. The process of coding collates the diversity of the surveyed set of papers through the formation of smaller ***codes***. We refer to each paper included within the scope of the survey as a ***research unit*** (RU), i.e., a publication (***excluding*** surveys) in conference proceedings and journals. Codes must have the following characteristics.

1. They must be semantically rigorous i.e., the meaning a code represents must be clear and use (application) of the code must be unconfusable.
2. They must be universally applicable across RUs, i.e. they must provide a uniform means of dissecting publications. Use of more than one coding system (i.e. two or more non-universal coding systems) may create a split in the coded data with incomparable parts across the split.

### How are codes formed?

We satisfy these two requirements through the elemental coding method of ***structural coding***. Structural coding poses ***a series of questions relevant to the inquiry in hand*** and is well suited to any problem which can be described using a standardized set of questions. We deconstruct the problem of literature review into a standardized question-and-answer protocol that can be directly converted into a structural coding approach. The questions are the following:

1. What is the problem which the researcher(s) saw as an opportunity for study?
2. What approach(es) did the researchers take in an attempt to solve the problem? Or: *how did they go about it … what did they* ***do***?
3. What development(s) and/or contributions derive from the researcher(s) work?.

The answers to these questions must emerge from the content of the publications (the RUs). Therefore, we may encode the RUs using the observation that these have three common manifest properties. The corresponding structural codes are:

1. **P**roblems (or challenges)
2. **A**pproaches
3. **D**evelopments (or contributions)

We refer to this protocol as the ***PAD review protocol***. Each RU (paper) is mined for:

1. “the problem which the researcher(s) saw as an opportunity for study” (P-codes),
2. the “approach(es) … [taken by] the researchers … in an attempt to solve the problem” (A-codes) and
3. the “developments(s) and/or contributions deriv[ing] from the researcher(s) work” (D-codes).

(quoted text is taken from the questions enumerated above).

**Fig. 1:**
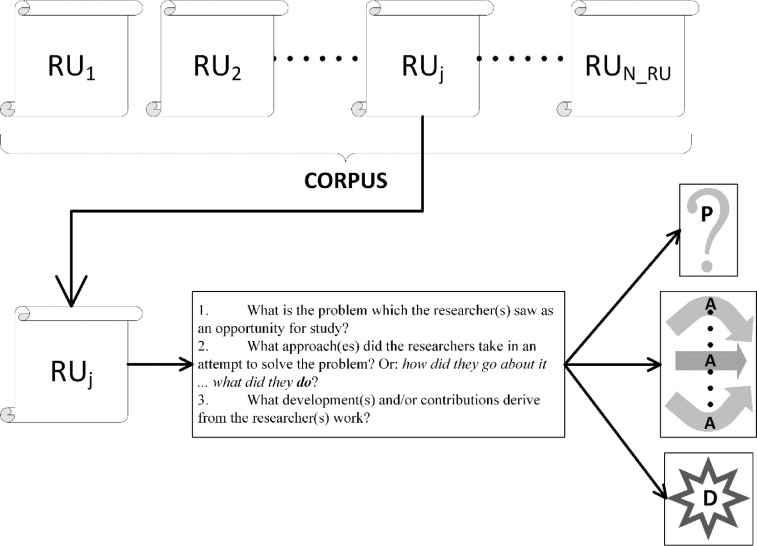
Each paper (RU) in the corpus is addressed using three research questions.

A representation of the concepts in the process of moving from RU to codes is shown in Fig. 1.

### Applying the structural code: identifying problems, approaches and developments

We now express a generalized understanding of the reasoning we follow to identify the problems, approaches and developments. In terms of thematic analysis, this is the role which an expert would play to transform the raw data into codes. This applies a first abstraction to the RUs:

• from detail specific to the RU
• to abstractive problem-, approach- and development-codes

that meaningfully represent the individual RUs. Succinctly, the role may be thought of as a ***parsing*** one.

#### Problem codes

Research is rooted in “the problem which the researcher(s) saw as an opportunity for study”. Key techniques in identification of the problem include:

• a focus on the abstract and the introduction,
• making a high-level summary, e.g., by answering questions such as:
  ○ what is this paper trying to solve?
  ○ what is this paper concerned with?
• recognition of revealing phrases like "in this paper" or "in this work", therefrom harvesting the authors’ explicit claims on the identity of the problem tackled.

The result of application of these techniques is a set of ***problem codes*** (P-codes). When the scope of a survey is relatively narrow, the problems in the set (i.e., the set of P-codes) may not be fully independent of one another. They may diverge from one another only as ***aspects*** (we could also say that they are ***derivatives***) ***of a core challenge***. In such a case, each RU (each paper) would be rooted in this core challenge, but the derivative problems (which are represented by the P-codes) addressed differ from one RU to another.

#### Approach codes

***Approach*** is used in the sense defined by the Oxford English Dictionary as “*figurative. A way of considering or handling something, esp. a problem*.” This meaning is broad yet it is purposely cited here to convey some of the depth of the difficulty we faced in assessing whether an observation candidate as an approach should be included (or not). Key techniques in identification of the approach include:

• a focus on the method section
• ***choices*** that focus technique

Codes of this type are the hardest to extract from Rus, as they are heavily dependent on a good grasp of the research space. This difficulty is severe enough to count as a limitation of our method (see Section 11.1). We emphasize that the “way of considering or handling something” is never (we dare say) obtained through a single approach, but, rather, through an entire delta (fan-out) of component approaches that are combined to bring efforts to a yield: the development.

#### Development codes

***Developments*** include what are commonly referred to as contributions, but we can afford to broaden our scope for inclusion of less significant products of research. Therefore, we go beyond simply parsing Rus in search of the familiar “contributions of our paper” or “in this paper” phrases, and glean those useful bits that, taken alone, do not qualify as the scope of a paper.

## Categorization: clustering the codes

The act of coding is carried out as an intermediate step on the way towards ***categorization***. We first introduce our use of the concept of the ***node***. We then proceed to a detailed treatment of how, through iterations, a set of problem-, approach- and development-codes can be categorized.

### Nodes – our use of a graph theory concept to complement structural coding

We use the concept of ***node*** from graph theory. Nodes are obtained from codes, as follows. The product of mining an RU is one or more P-, A- and d-codes. For every code, the text of the code, which we label, say, as α, is associated with a numerical, integral co-attribute, which we label, say, as a, and both the text and number are added as attributes of a ***node***. The textual code and integer are ***attributes*** of the node. Each text-code – integer pair is encapsulated within a node. Since problem, approach and development are linked to one another in ***a causal chain, this causal relationship can be represented through linked nodes.*** Each such causal chain is represented by three nodes; thus, we call it a ***triad***. Our application of the node concept is beneficial for the following reasons:

1. An RU's collection of triads (of nodes) is a synthetic representation of the RU, that facilitates a good apprehension thereof.
2. Once a complete graph of the corpus has been compiled, these characterizing triads facilitate the task of locating this RU within the greater landscape of research.

**Fig. 2:**
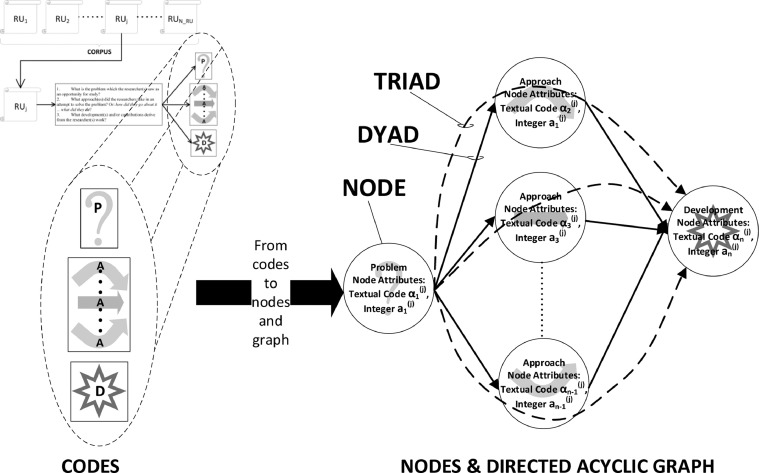
RU codes are transformed into nodes, which are then linked by dyads and triads in a DAG.

Figure 2 shows a summary of the process (from-codes-to-DAG) of transforming codes into nodes and use of the causality binding the problem, approach and development nodes. The figure illustrates one problem node linked to several approach nodes and the approach nodes converged onto a development node. This divergence (from problem to approaches) and convergence (from approaches to development) is common. A paper (RU) characterized by such a research mapping would tackle one problem through a complementary set of approaches, and make some headway in solving the problem, which we refer to as the development. While common, this is not the only possible research mapping. Other mappings we have found describe several developments in a single RU, while other mappings describe Rus that tackle several aspects of a root problem.

### The iterative process of categorization

For every RU in the corpus, we iterate through a number of steps to categorize a code. Given an RU, a set of ***problem, approach and development*** codes is collected and added to a pool of ungrouped codes. Consider any one of these codes, of some type (either P, or A, or D), with label say, α, and co-attribute, say, a.

Seek a pre-extant code (of the same type, i.e., P/A/D) ***nearest in meaning***, say code β, with numerical, integral co-attribute (say) b. If there is no neighbor (in meaning) sufficiently close to group α with, then this iteration of categorization terminates for code α and it is kept within the pool of ungrouped (orphan) codes. Else, if β is ***not*** already grouped within a category, then create a category with ***proximating meaning*** Γ ***common to both*** α ***and*** β, and with numerical, integral co-attribute C (upper case, to distinguish this as a category). Replace numerical co-attribute b by C.1 and replace a by C.2 Add both the text Γ and number C as attributes of a ***category-node***.

On the other hand, if β ***is*** already grouped within a category, revise (as necessary) the scope (and therefore, the text) of the proximating meaning to take α into account. If the proximating meaning (i.e., Γ) cannot be reasonably revised to include code α, either spawn a new category, containing codes α and β; or keep α as an orphan (i.e., without a grouping category), within a pool of ungrouped codes.

Note that:

• ***we refer to the proximating meaning as the category code;***
• if β had already been grouped within a category, its numerical co-attribute would already be of the float type (i.e., include a decimal separator), as it would have been previously transformed from numerical integer b to some float type that includes the digit(s) of its grouping category.

**Fig. 3:**
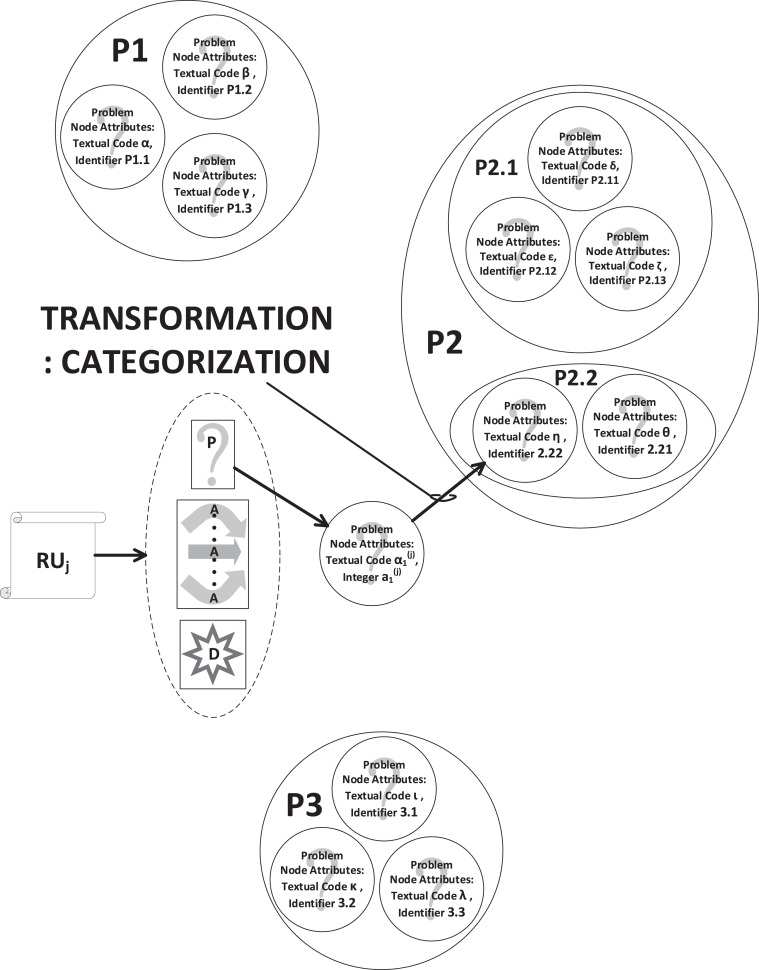
Meaning of an extracted code is compared with existing codes and categorized with its nearest neighbors.

The category code describes the salient meaning common to all codes grouped under it. It is the abstraction that sacrifices some detail for the sake of facilitating a holistic view of the research space. The resulting categorization is conducive to (a) assimilation by a viewer, as well as (b) further rationalization. In Fig. 3, we show, as an example, how categorization of the problem code of paper RUj has concluded with the addition of node P2.22 (this is not the same as the node in the data set included with our work) to sub-category P2.2 of category P2.

## Node attributes

The process of categorization results in a set of problem-category-, approach-category- and development-category-nodes. Each type of category-node groups nodes of the same type (P/A/D). In this section, we formalize the description of the attributes of the individual nodes and those of the category-nodes. The attributes, described below, of the individual nodes are those of nodes that have been categorized (i.e., the post-categorization attributes), as indicated (below) by the float type of the alphanumeric attribute of the node.

### Node attributes

1. A short textual description – the ***code*** – of the observed problem/approach/development and
2 A unique alphanumeric identifier (a ***label***) of the form P/A/Dx.yz. In this labeling scheme.

a. P, A and D represent the structural codes, b. x is a single digit that represents the observed problem/approach/development (P/A/D) category, c. y is a single digit that represents an observed sub-category of the P/A/D category, if any such sub-category is observed, and d. z is a single digit that represents the individual, observed problem/ approach/ development.

We chose the notation x.yz instead of x.y.z to type the variable represented by this identifier as a numeric float type.

### Category-node attributes

1. A short textual description – the ***category code*** – of the observed P/A/D category;
2. A unique alphanumeric identifier (a ***label***) of the form P/A/Dx. In this labeling scheme,

a. P, A and D represent the structural codes, b. x is an integer that represents the observed problem/approach/development category.

1. a set of member nodes.

Where major sub-clusters need to be identified, we extend the labeling to P/A/Dx.y,where y is a second integer representing the sub-cluster. In these cases, the (member-) node identifier takes the form P/A/Dx.yz.

## Graphical maps of the research space

The workings of the method culminate in the production of several graphic devices, which we describe below.

### The causality DAG

The P-, A- and d- category-nodes are linked according to the triads mined from the RUs, to produce a graphic device which may be tersely and aptly referred to as a ***directed acyclic graph (DAG) of causality*** - the causality DAG. This graphic device is an important part of the product of our method. It shows a bird's-eye view of the dynamics of research in our chosen scope.

In particular, the DAG is an encoding of surveyed research units, that strives to relieve a profile not only of current knowledge (the ***developments***) but also of what has been found a fruitful pursuit (the ***approaches***) thereof. This is obtained through the relationships that are illustrated in the DAG between the problems addressed, the approaches to solutions, and the knowledge obtained in pursuit of solutions.

The DAG may show links between ***category-nodes***, rather than individual nodes, to minimize clutter and improve readability. Such a reduction is strongly dependent on truly representative categorization (see Section 5), through groupings meaningful to the survey's scope. In this form of the DAG, the inter-category links are ***aggregators***: they include all links between any two nodes within their respective categories. ***Line thickness*** is an excellent way to represent the size of the set of aggregated links. We present Fig. 5 from our case notes for further detail. Note that the complexity of this graphic can be reduced arbitrarily by establishment of a lower limit on the size of the aggregated links to display. For example, it might be decided to focus only on those links that are of a size greater than the 50th percentile of thicknesses. In this way, the thinner lines are removed from the graphic. This selectivity can be exercised within the framework of a judicious inspection of the graphical results of the survey.

### Triads graphic

We also highlight triads. Triads are represented by lines passing through a single combination of a single problem, a single component of approach and a single development to which the approach (component) led (not necessarily on its own; indeed, rarely so). The triads graphic identifies the dynamics of research at a glance, indicating the most highly used triads by line thickness.

As with the DAG (and for the same reason), the triads graphic may show causal bindings between category-nodes, rather than individual nodes. Each triad is a serial connection of one link from a P-category to an A-category, and one link from the latter A-category to a D-category. Each triad is illustrated with a bend where links meet on A-category nodes. As with the DAG, we present an example (Fig. 6) and point out that complexity can be arbitrarily adjusted.

### P-A dyads graphics: challenges (P) and associated approaches (A)

The causality DAG's section showing P-A category links is partitioned into a set of P-A dyad-graphics. Each such graphic illustrates the set of all approach components that have been used to tackle a particular problem category. The P-A dyads graphics complement the causality DAG and the triads graphic by a focus on how individual challenges have been tackled in published research. Each P-A dyads graphic provides, from the perspective of a specific challenge, the same information on frequency (of applied approaches) as the (global) causality DAG. We present two examples of this type of graphic in the case notes (Figs. 7 and 8).

### Taxonomies of problems, approaches and developments

Within each of the three divisions (i.e., problems, approaches and developments) of categories, frequency of occurrence of categories is expected to communicate meaningful information about the state of the art. Furthermore, within each category-division, it may be possible to find relationships between categories that convey additional meaning and encourage structural formations that gather the categories into ***taxonomies***. For example: if two categories of approaches are proximal in meaning, a super-category might be formed that abstracts the differences in meaning between the two and represents them both from the perspective of the common, salient meaning.

## Statistics

The frequency of occurrence of aspects of data collected is examined here. We combine the structural codes in various ways in order to obtain useful statistics for a quantitative grasp of the field. In the following sub-sections, we suggest and describe how these statistics can be collected from the data.

### Frequency of occurrence of a challenge category in RUs

We start by proposing the following simple metric.

(1) $$F_{P_{k}}=\frac{\sum_{j=1}^{N_{RU}}P_{k}^{\left(j\right)}}{N_{RU}}$$

where:

1. $P_{k}^{\left(j\right)}$ is a binary variable that represents the presence (or lack thereof) of a problem in category $P_{k}$,
2. within a single RU $RU_{j}$ over the corpus of $N_{RU}$ unique RUs.

This simply indicates the number of times in which a challenge-category appears within the RUs in the corpus, as a fraction of the total number of RUs. The numerator of $F_{P_{k}}$ is incremented once by $RU_{j}$ for a given $P_{k}$ if a problem in this category is tackled in $RU_{j}$.

### Research interest: frequency of occurrence of a challenge category among all occurrences of challenge categories

Here, we define ***research interest***, denoted by $R_{P_{k}}$, in a given challenge (problem) category $P_{k}$, as its frequency of occurrence within the set of all the challenges tackled in all research units. It is computed as the total number of times in which problems in category $P_{k}$ have been tackled in RUs, as a fraction of the sum of the total number of times in which (problems in) all observed challenge categories have been tackled. We suggest this as a metric of the attention, or research interest, which this challenge is receiving. The numerator of $R_{P_{k}}$ is incremented once for $RU_{j}$ for a given $P_{k}$ if a problem in this category is tackled in $RU_{j}$.

(2) $$R_{P_{k}}=\frac{\sum_{j=1}^{N_{RU}}P_{k}^{\left(j\right)}}{\sum_{i=1}^{N_{P}}\sum_{j=1}^{N_{RU}}P_{i}^{\left(j\right)}}$$

where:

1. $P_{k}^{\left(j\right)}$ is a binary variable that represents the presence (or lack thereof) of a problem in category $P_{k}$,
2. within a single RU $RU_{j}$ over the corpus of $N_{RU}$ unique RUs,
3. with a total of $N_{P}$ unique, identified challenge/problem categories.

Note that:

(3) $$\frac{F_{P_{k}}}{R_{P_{k}}}=\frac{\sum_{i=1}^{N_{P}}\sum_{j=1}^{N_{RU}}P_{i}^{\left(j\right)}}{N_{RU}}$$

This ratio is a constant; therefore, both $F_{P_{k}}$ and $R_{P_{k}}$ have identical distributions, but the statistics differ. Specifically, the ratio $\frac{F_{P_{k}}}{R_{P_{k}}}$ is the average number of challenges tackled per RU.

### Metric of observed approach diversity: weighted challenges

We measure the diversity of approaches through which a challenge is tackled. The set of all unique problem-approach (PA) pairs (dyads) in the triads is collected first. Therefore, a particular PA dyad is counted once, regardless of the number of occurrences of that dyad. Then, for each problem, we add up the total number of dyads within which that problem is found. We suggest a normalized diversity metric, $W_{P_{k}}$, as follows:

(4) $$W_{P_{k}}=\frac{\sum_{j=1}^{N_{PA}}P_{k}^{\left(j\right)}}{\sum_{i=1}^{N_{P}}\sum_{j=1}^{N_{PA}}P_{i}^{\left(j\right)}}$$

where:

1. $P_{k}^{\left(j\right)}$ is a binary variable that represents the presence (or absence) of a problem category $P_{k}$,
2. within a single P – A dyad $PA_{j}$, over the set of all $N_{PA}$ unique P – A dyads within the corpus,
3. with a total of $N_{P}$ unique, identified challenge/problem categories.

### Frequency of occurrence of an approach category among all occurrences of approach categories

Unlike problems in challenge categories, approaches within an approach category do not, most commonly, mutually exclude one another. Therefore, the count of occurrences of an approach category may be incremented more than once per RU and thus a metric like $F_{P_{k}}$ lacks a good normalization basis. As it is useful to learn how widely exploited an approach is, a different normalization basis must be selected. We therefore use a metric somewhat similar to $R_{P_{k}}$, i.e., frequency of occurrence of an approach category in all RUs, among the set of all occurrences of approach categories in all RUs. We obtain the metric $R_{A_{k}}$, as follows:

(5) $$R_{A_{k}}=\frac{\sum_{j=1}^{N_{RU}}\sum_{m=1}^{\left|A_{k}\right|}A_{k_{m}}^{\left(j\right)}}{\sum_{i=1}^{N_{A}}\sum_{j=1}^{N_{RU}}\sum_{m=1}^{\left|A_{i}\right|}A_{i_{m}}^{\left(j\right)}}$$

where:

1. $A_{k_{m}}^{\left(j\right)}$ is a binary variable that represents the presence (or lack thereof) of ***approach*** $A_{k_{m}}$,
2. where $A_{k_{m}}$ is a member of ***approach category*** $A_{k}$, of cardinality $|A_{k}|$,
3. within a single RU $RU_{j}$,
4. over the corpus of $N_{RU}$ unique RUs,
5. with a total of $N_{A}$ unique, identified approach categories.

Note that occurrence of category $A_{k}$ within an RU is counted as many times as its members $A_{k_{m}}$ appear in the RU.

### Metric of utility of an approach: weighted approaches

We also analyze approaches in terms of their utility, i.e., how useful they are in the overall motion between problems and developments, and denote this metric as $U_{A_{k}}$. The numerator is incremented each time a particular approach is a component of a triad within an RU. Therefore, a single RU may increment the metric several times. The utility metric of a specific approach $A_{k}$ is the normalized metric:

(6) $$U_{A_{k}}=\frac{\sum_{j=1}^{N_{RU}}\sum_{l=1}^{N_{triads_{RU_{j}}}}A_{k}^{\left(l\right)}}{\sum_{i=1}^{N_{A}}\sum_{j=1}^{N_{RU}}\sum_{l=1}^{N_{triads_{RU_{j}}}}A_{i}^{\left(l\right)}}$$

where:

1. $A_{k}^{\left(l\right)}$ is a binary variable that represents the presence (or lack thereof) of approach category $A_{k}$,
2. within any of the $N_{triads_{RU_{i}}}$ triads in a single research unit $RU_{j}$,
3. over the corpus of $N_{RU}$ unique RUs,
4. with a total of $N_{A}$ unique, identified approach categories.

We emphasize that a single research unit may be described by several such triads that include approach $A_{k}$.

### Frequency of occurrence of categories of development

Development statistics are distributed thinly unless developments are categorized. However, when grouped into meaningful clusters (categories), conclusions can be drawn about the frequency with which developments take place in sub-spaces of this research space. We obtain $R_{D_{k}}$, the normalized frequency of occurrence of categories, as follows:

(7) $$R_{D_{k}}=\frac{\sum_{j=1}^{N_{RU}}\sum_{m=1}^{\left|D_{k}\right|}D_{k_{m}}^{\left(j\right)}}{\sum_{i=1}^{N_{D}}\sum_{j=1}^{N_{RU}}\sum_{m=1}^{\left|D_{i}\right|}D_{i_{m}}^{\left(j\right)}}$$

where:

1. $D_{k_{m}}^{\left(j\right)}$ is a binary variable that represents the presence (or lack thereof) of ***development*** $D_{k_{m}}$,
2. where $D_{k_{m}}$ is a member of ***development category*** $D_{k}$, of cardinality $|D_{k}|$,
3. within a single RU $RU_{j}$,
4. over the corpus of $N_{RU}$ unique RUs,
5. with a total of $N_{D}$ unique, identified development categories.

Thereby, a prospective researcher is guided through grounded insight into works covering this space.

## Deriving themes

The concept of a theme is consistently described as a ***pattern*** that emerges from the raw data; see, e.g., [4, p. 82] and [3, p. 4]. The PAD enhancement of structural coding facilitates thematic analysis by accentuating themes through the abstractive function of causally-bound categories of codes. Both quantitative and qualitative aspects of analysis are possible.

### Quantitative analysis

The ***results*** of application of the PAD method are powerfully conducive to a quantitative aspect of thematic analysis:

1. The frequency of individual categories (i.e., whether problem-, approach- or development-categories) is itself meaningful;
2. it is possible to attempt an interpretation of the frequency of occurrence of pairs (***dyads***) of problem – approach categories, and
3. the frequency of a triad within the overall set identified is highly representative of the state of the art. We find it more summative to observe triads of category-nodes than triads of individual nodes, as the latter disperse frequency of occurrence too thinly. Categories act as bins that aggregate frequency usefully.

### Qualitative analysis

Qualitative analysis inheres in the very ***processes*** of coding and categorization, and it is further accentuated by the causal linkages between the categories of the structural code. The observed categories, dyads, triads and their relative frequencies are fertile grounds for ***grounded reflection*** about the state of research. Some examples are given in the context of the case notes (Section 10.4).

This culminates our thematic analysis.

## Case notes

We have used the PAD method during a survey of research into power modeling and measurement in virtualized environments. The surveyed body of papers was gathered from the ACM, IEEE and other sources (the “corpus”). The corpus is our raw qualitative data.

### Paper selection criteria

Every surveyor will decide on the relevance (in/out of scope) of a paper by considering certain criteria. A generally valid criterion is to exclude other surveys from the corpus, as a survey is not itself comparable with the works within its scope. This does not exclude surveys from consideration, since a prospective surveyor would be well advised to learn about what ground other surveyors have covered and results obtained from their coverage. However, the surveys do not themselves constitute raw qualitative data: they contain results obtained from the processing of raw data.

**Fig. 4:**
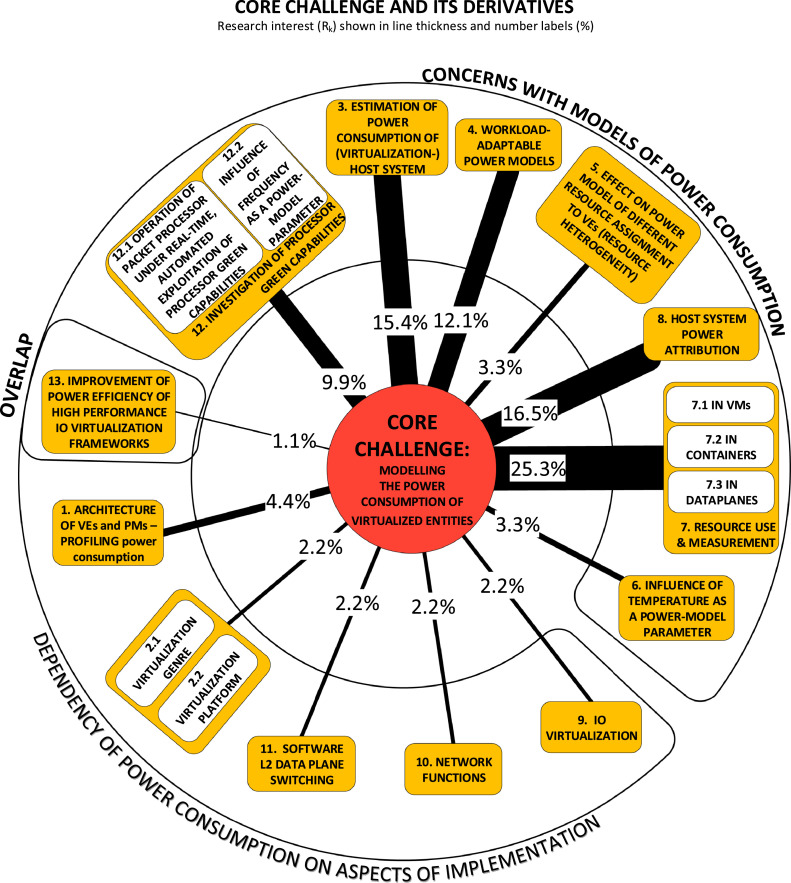
Categories of challenge that emerged as a result of structural coding.

In our case, the criterion that proved most effective in sorting RUs into relevant or irrelevant was the challenge undertaken (the P-node). It seems fair to extrapolate this observation to surveys in general, or at the very least, to a major category of surveys. Many surveys of the state of the art in some field of a discipline are oriented towards the progress achieved in tackling the field's challenges. Hence, the generalization we suggest would hold for this category of surveys. An illustration of how the field of study emanates categories of challenges (the P-category-nodes) is shown in Fig. 4.

### Identifying codes and categorizing them

Problem codes are frequently identified using the abstract and certain key phrases like "in this paper", or "this paper", or "in this work". As the number of RUs perused increase, nuances start emerging and codes that at first appeared to be somewhat distinct are recognized as factually indistinguishable, even before actual categorization begins. These codes are assigned a number and integrated with their numbers in nodes (node = code + number).

As with problem codes, the abstract of an RU is a good source of development codes. At least some development codes are usually self-evident (i.e., ***semantic***) here, since researchers are keen to point out their primary contribution(s) (developments) and hold on to readers’ notoriously volatile attention. However, thorough harvesting was only obtained with an organic growth in familiarity with the field, as contributions that were less conspicuous or weighty started to emerge as more RUs were perused. These secondary contributions were distributed throughout the length of papers. Therefore, the pace of harvesting development codes was slower than its problem-codes counterpart, as their transparency, number and distribution were less favorable.

Most problematic were the approach codes. These require a difficult movement from the general meaning (“a way of considering or handling something, esp. a problem”) to the domain-specific meaning. A useful generalization is that these codes describe the empirical setup, and RU sections titled “method” or “methodology” are good sources of codes. Indeed, this would be the “way of … ***handling*** something.” However, there is the rather ***latent*** aspect of researchers’ “way of ***considering***… a problem”. For example, we observed that models developed are at least in part the result of an approach towards modeling, and justification of the selected form of power model is strongly dependent upon the inputs and parameters of operation.

Among the files in our study's dataset [10]:

• we cross-reference a comprehensive list of harvested codes(labels) and node numbers, and
• we present all node triads harvested from the corpus.

Categorization of codes proceeded as described in Section 5, with P-, A-, and D-nodes handled separately. While the final categorization of each code type required several iterations over the full set of codes, it was a comparatively lightly taxing endeavor. The process of coding directly led our coding work through the considerations that drive categorization: inspection, comparison, contrast, etc., all while noting down codes.

### Graphical maps

**Fig. 5:**
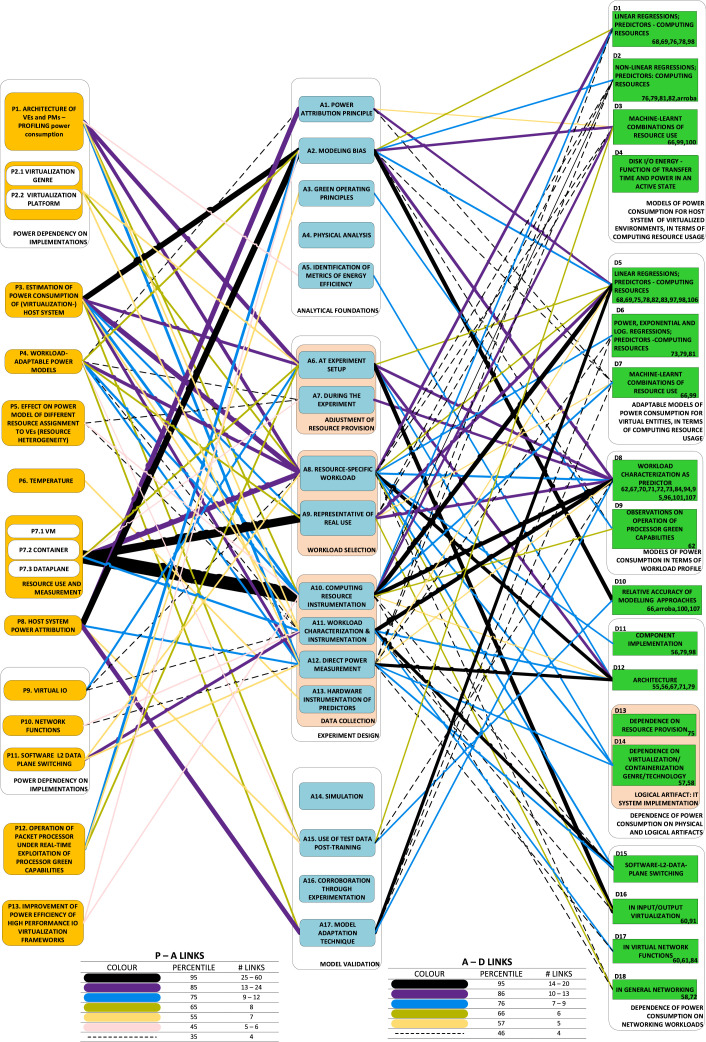
The directed acyclic graph (DAG) linking the category nodes.

The causality DAG derived from our study is shown in Fig. 5. The concern with problems in category P7 (resource use and measurement) stands out in the DAG. The primary category of approaches taken to tackle this problem is A10 (through instrumentation of computing resources).

**Fig. 6:**
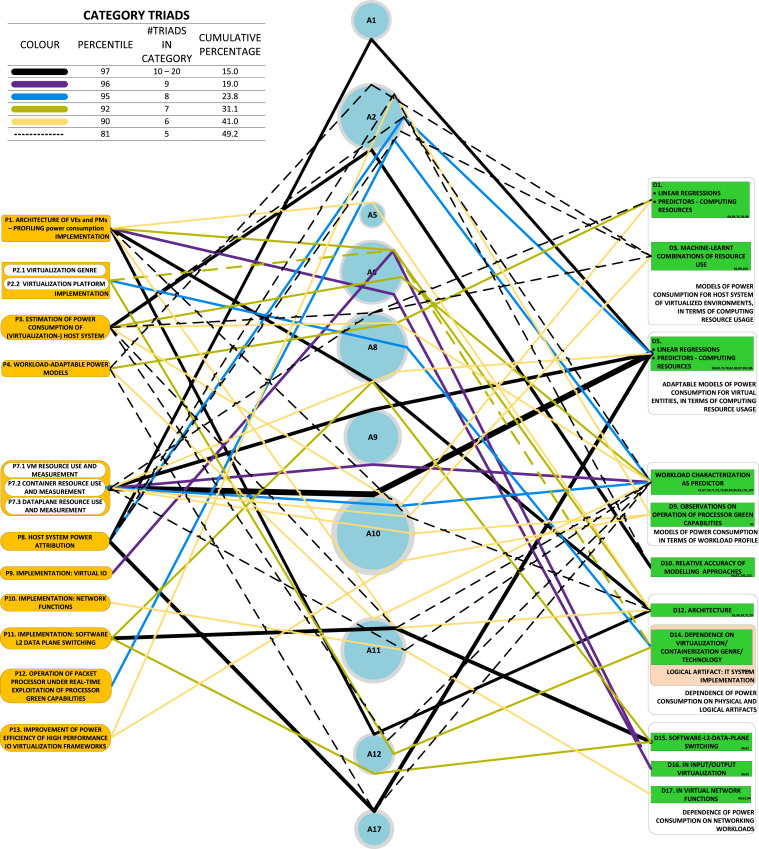
The triads graphic throws prominent research dynamics into sharp relief.

The triads graphic derived from the study is shown in Fig. 6. In this graphic, it can be seen that the development that has emerged most frequently out of the P7-A10 dyad is that of models that regress use of computing resources onto a linear relationship with power consumption.

**Fig. 7:**
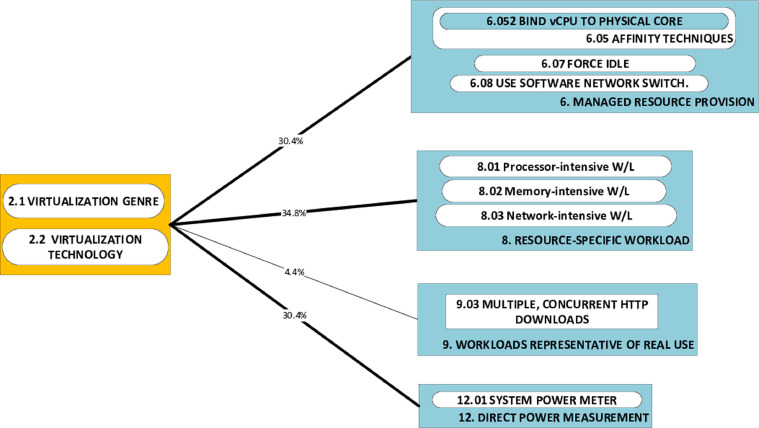
Frequency of occurrence of approaches to tackling P2, shown in line thickness & as percentage.

**Fig. 8:**
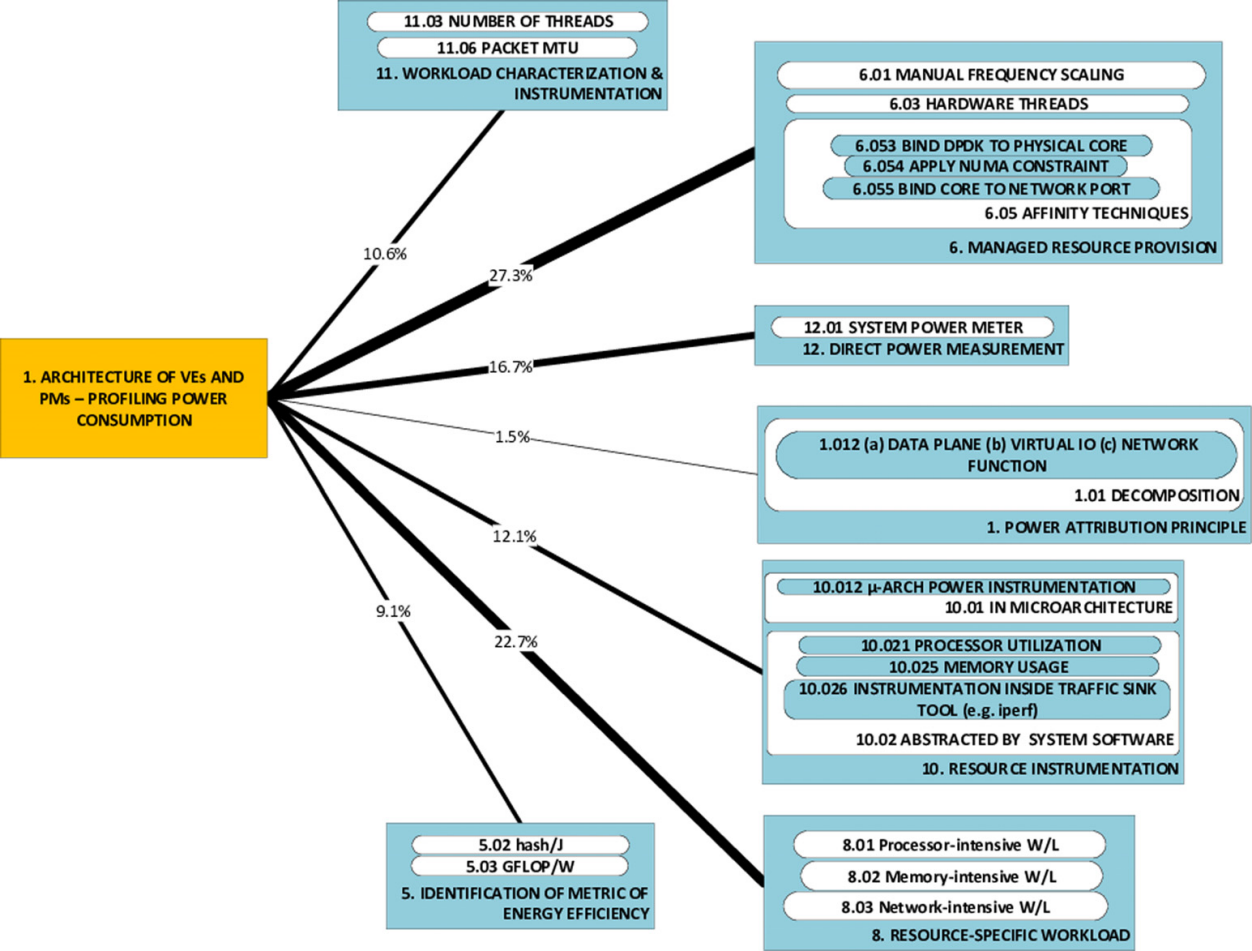
Frequency of occurrence of approaches to tackling P1, shown in line thickness & as percentage.

Finally, we include herein two examples of dyad graphics from our study. We have chosen direct inclusion of these graphics as they are the least dense of all the devices and thus well suited to serving as a handy example Fig. 7. shows that the challenge of obtaining broad indications of the effect of architecture on power consumption is tackled through a wide variety of approaches Fig. 8. shows that cross-comparison of power consumption of virtualization genres and technologies (the challenge) has been primarily tackled using workloads that target specific resources (34.8%), rather than workloads that represent real use (4.4%).

Several other graphic devices were compiled using this method's products. It has proven to be well suited to the compilation of taxonomies of challenges, approaches and developments in the surveyed field.

### The emergence of themes

**Fig. 9:**
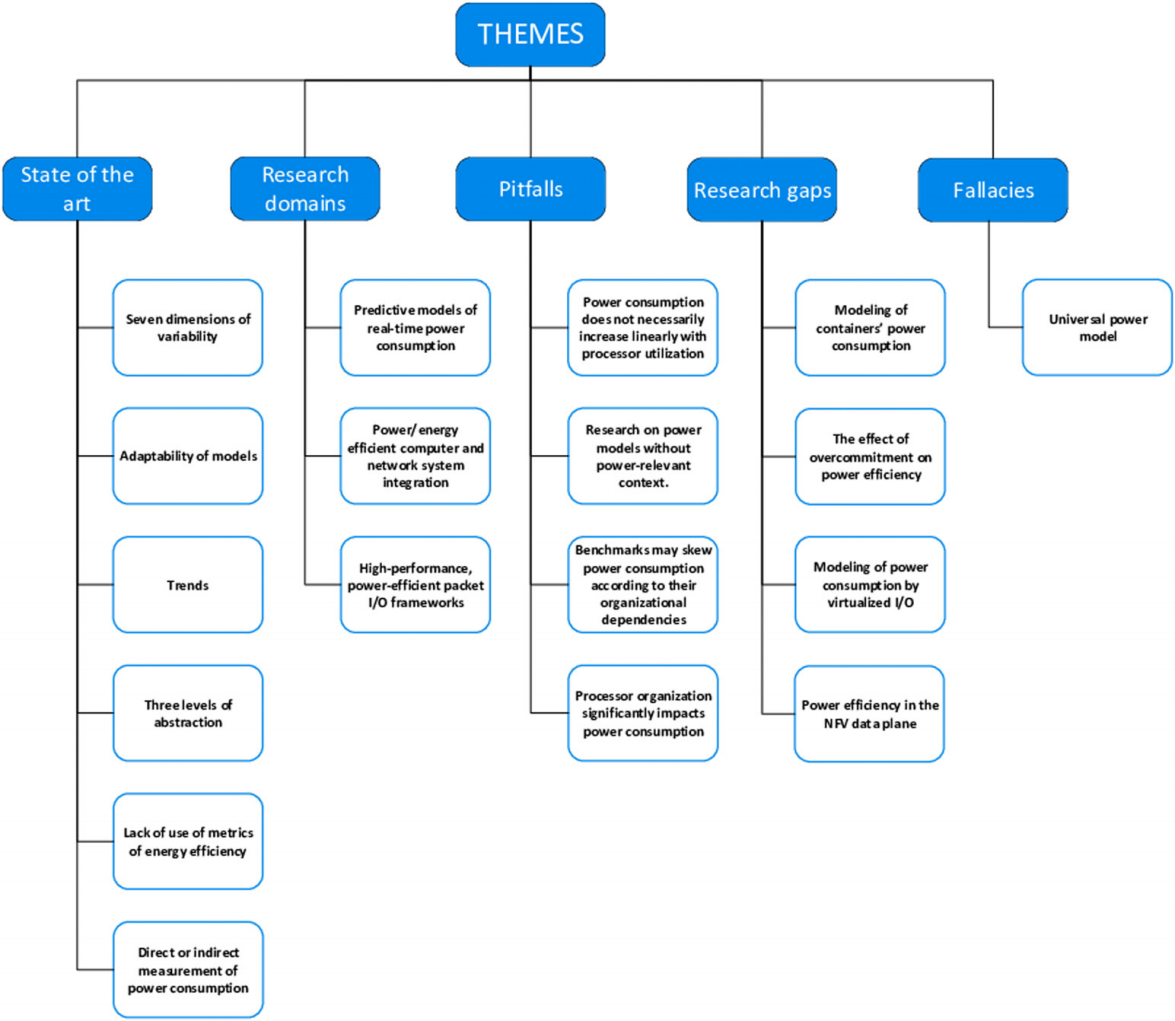
An overview of the qualitative aspect of thematic analysis of the research space.

Our knowledge of the surveyed domain developed as we progressed in parsing RUs and discussing the coding and categorization thereof. These processes - coding, categorization, linking and discussion - led to a thorough qualitative analysis, which was itself amenable to classification. Thus, we were able to give a bird's-eye view (see Fig. 9) of:

• The state-of-the-art, including trends in research;
• Research gaps;
• Pitfalls and fallacies, and
• Domains which demand research into models and measurement of power consumption.

## Conclusion

### Benefits and limitations

Thematic analysis with structural coding provides at least four distinct benefits.

1. It facilitates ***the discovery of patterns*** in research, through the causality DAG. The causality DAG is a map that illustrates a structural encoding of primary research sources. This is a robust base for the development of a set of themes that represents the ongoing effort of discovery within the field of study. Patterns may be found in the horizontal dimensions of the DAG. A pattern in the horizontal dimension can consist of a PAD triad that recurs several times in the data. It can also consist of a group of triads that share both the P-category-node and the A-category-node. Such a group of triads comprises a variety of developments that arise out of the same approach to the same problem. A looser but nonetheless interesting grouping comprises those triads that share the P-category-node. This would be useful to a researcher seeking to learn how others who have addressed the same problem.
2. It facilitates ***the evaluation of the novelty of research proposals***. Whether the proposal has reached the stage of problem identification or selection of approach, the DAG is a useful tool in the assessment of the likelihood of developing successful research out of the proposal. This follows because inspection of the map leads to an indication of the density of research in and near the space under consideration for research.
3. Unlike simpler, ad hoc surveying, the processes used by this method are open to scrutiny through identifiable and tangible proceedings. Whilst still a subjective (see limitations) method, its workings are more amenable to a reader's analysis than other, less open techniques.
4. Structural coding leads the surveyor to process his/her corpus systematically. The generalizable, parsing technique reduces the verbosity of text to a regular set of codes that aptly and succinctly describe the unit of research.

Two limitations have emerged.

1. ***Clustering coarsens resolution.*** The causality DAG links category-code-nodes. This results in an apparent linkage between any node within one category-node and any node within the category-node at the other end of the link. However, this is not necessarily reflected in the RUs we have mined and is a result of the loss of resolution that accompanies clustering. The effects of this generalization can be mitigated by ensuring that the clustering notion has narrowly defined meaning. Such narrowness in meaning reduces the scope for interpretative error.
2. ***The need for multiple iterations.*** Identification of codes is limited by the reviewer's breadth of vision of the field under review. Since the work may be carried out with the very purpose of gaining a broad view, it may seem that a Catch 22 is embedded in the method. This is not expected to be as frustrating as it may appear. It is expected that a reviewer has some background within the field. The same ability used to search the field may be exploited during an initial coding ***iteration***. As the review proceeds, the reviewer's breadth of vision expands and the set of codes is grown through refinement of existing codes and addition of new ones. The limitation may be experienced in any of the P-, A- and D- categories. In particular, during this survey, perception of approaches improved as more RUs were parsed. Indeed, this progression is recognized as part of the labor of research: it is partly undertaken during an initial familiarization [4, p. 87] and partly as a cyclical re-evaluation of RUs in the light shed by discovery of new codes [7, p. 8].

### Method summary

**Fig. 10:**
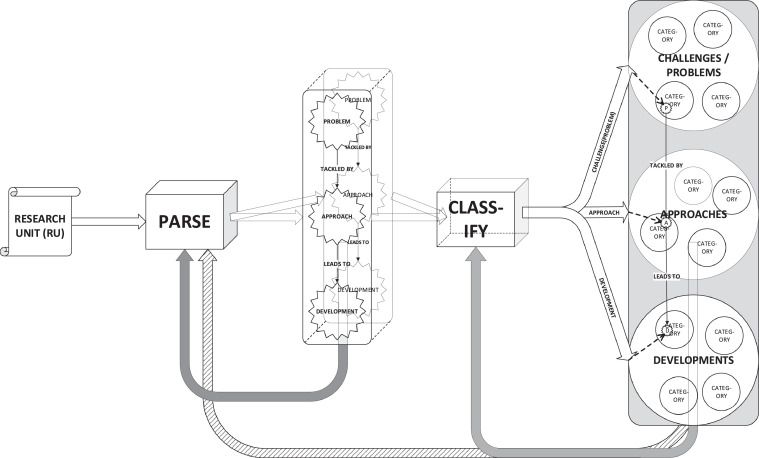
A systematic approach to critical review of a corpus of literature.

Using thematic analysis with structural coding, a literature review can process a diverse corpus of RUs to produce a succinct, graphical, numerical and analytical representation of the research space. Proceedings may be divided into the following separate tasks. Tasks 1–5 are illustrated in Fig. 10.

1. Papers are mined (***parsed***) for triads of problems, approaches and developments, using the review protocol described in Section 4.2.
2. The codes are clustered around their structural codes, while keeping the links between the codes that organize them into triads.
3. The clusters are divided into a number of tightly-packed clusters. These clusters are identifiable as categories.
4. A category-node is obtained from of each such category, as described in Categorization.
5. The category-nodes are linked sequentially (horizontally) according to the mined triads to form a causal chain that proceeds from problem to development. The complete set of triads forms the research space's directed acyclic graph of causality (causality DAG).
6. Quantitative analysis is possible through the suggested metrics, which provide statistical information on the field. These are complemented by the causality DAG, triads graphic, P-A dyads graphics and taxonomies, which combine to give a multi-faceted profile of the field.
7. Qualitative analysis is facilitated through the means for grounded reflection provided by the causality DAG and the quantitative analysis.

## Declaration of Competing Interest

The authors declare that they have no known competing financial interests or personal relationships that could have appeared to influence the work reported in this paper.

## Appendix Supplementary materials

Image, application 1

Image, application 2

Image, application 2

Image, application 2

Image, application 2
