# Supplementary material for: PAD: A graphical and numerical enhancement of structural coding to facilitate thematic analysis of a literature corpus
Source: MethodsX. 2022 Feb 15;9:101633. doi: 10.1016/j.mex.2022.101633 (PMC8891713; doi:10.1016/j.mex.2022.101633)

CATEGORY TRIADS

COLOUR	PERCENTILE	#TRIADS IN CATEGORY	CUMULATIVE PERCENTAGE
	97	10 – 20	15.0
	96	9	19.0
	95	8	23.8
	92	7	31.1
	90	6	41.0
	81	5	49.2

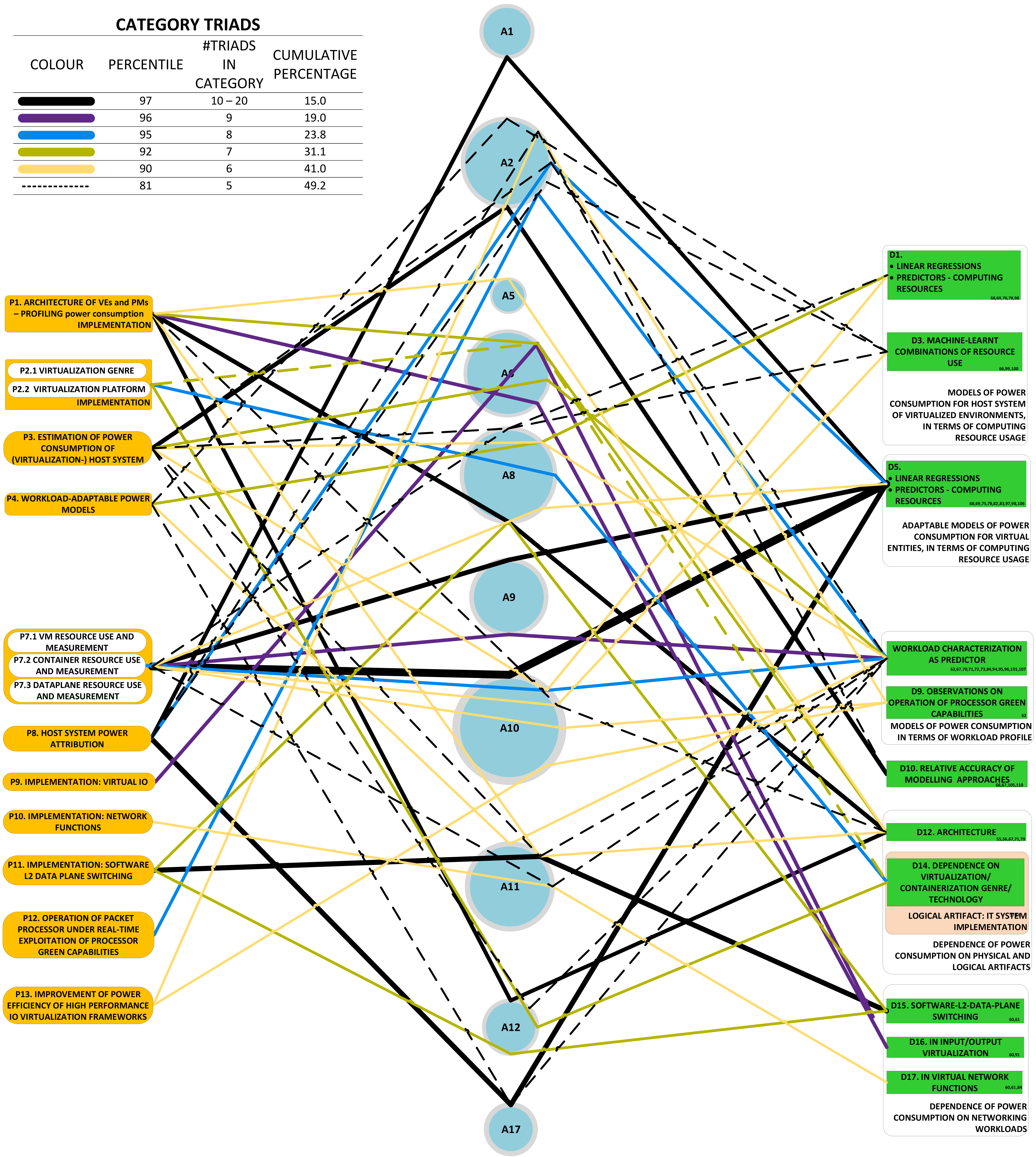

Supplement: Supplementary file 1 [file mmc1.pdf]
