# Supplementary material for: PAD: A graphical and numerical enhancement of structural coding to facilitate thematic analysis of a literature corpus
Source: MethodsX. 2022 Feb 15;9:101633. doi: 10.1016/j.mex.2022.101633 (PMC8891713; doi:10.1016/j.mex.2022.101633)

CORE CHALLENGE AND ITS DERIVATIVES

Research interest (R_k) shown in line thickness and number labels (%)

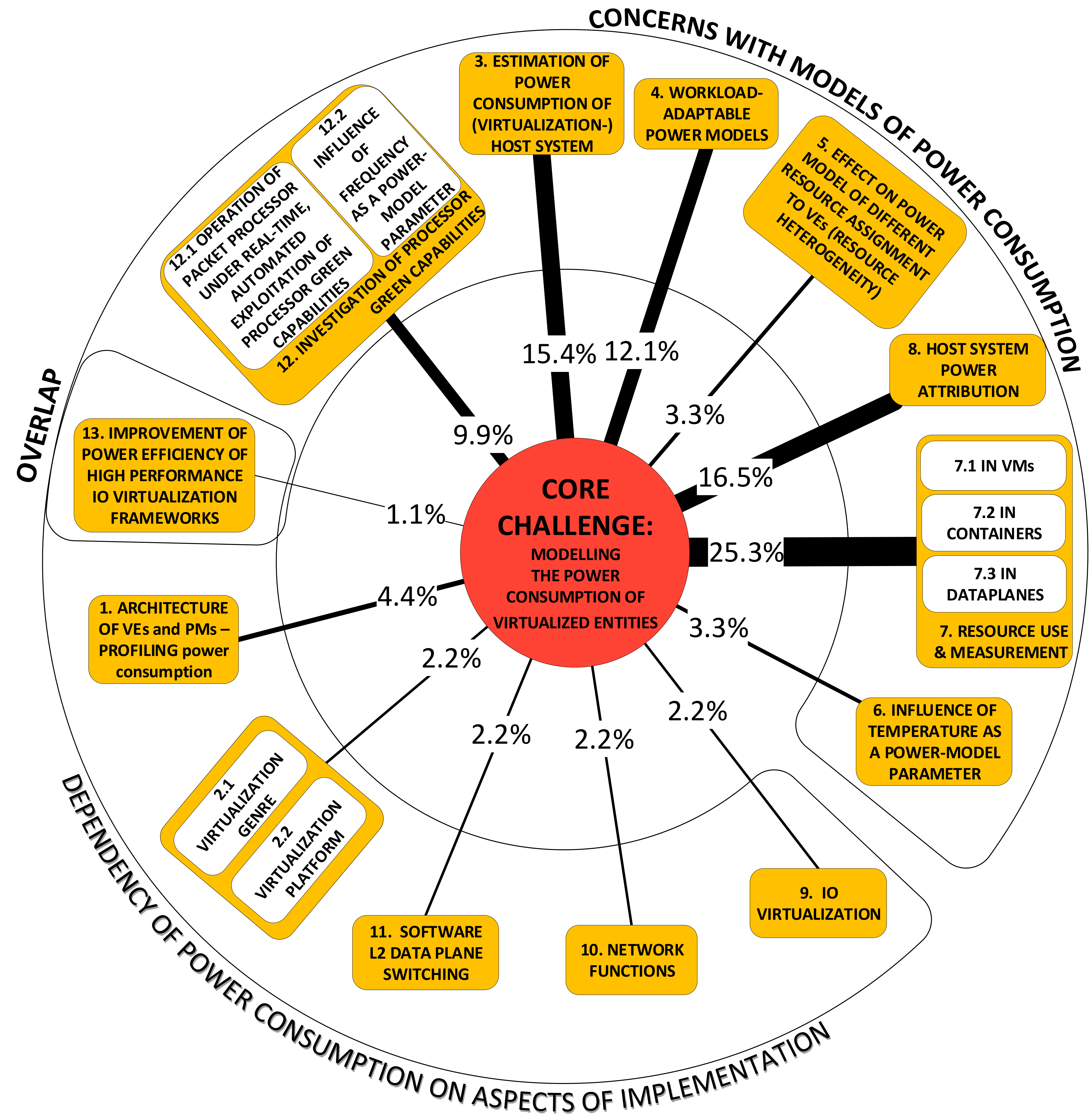

Supplement: Supplementary file 2 [file mmc2.pdf]
