# Supplementary figures and images for: PAD: A graphical and numerical enhancement of structural coding to facilitate thematic analysis of a literature corpus
Source: MethodsX. 2022 Feb 15;9:101633. doi: 10.1016/j.mex.2022.101633 (PMC8891713; doi:10.1016/j.mex.2022.101633)

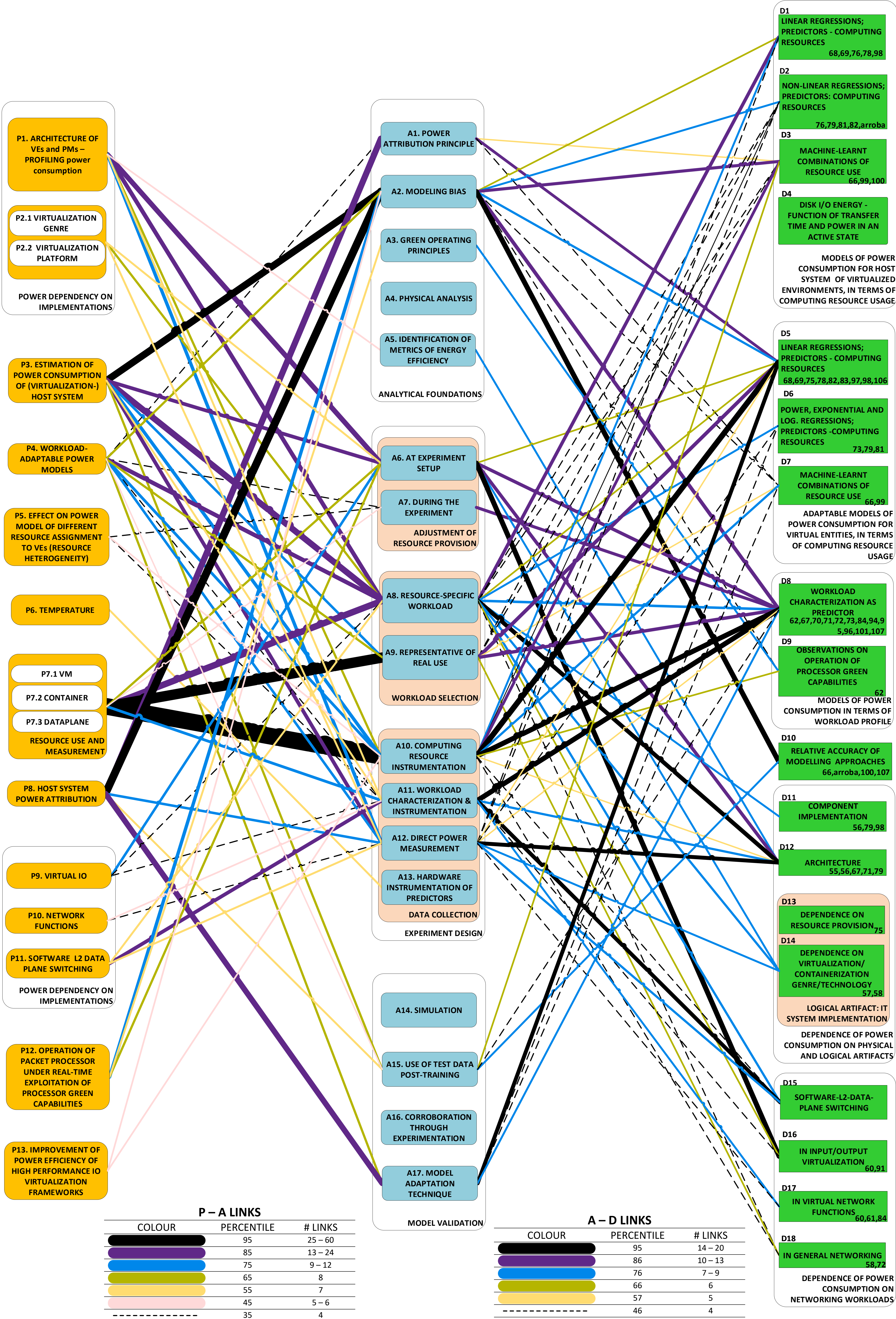

Supplement: Supplementary file 3 [file mmc3.pdf]
